# ETIOLOGIES OF INFECTIONS IN DIABETIC PATIENTS HOSPITALIZED AT BOUAKE UNIVERTY TEACHING HOSPITAL, CÔTE D’IVOIRE

**DOI:** 10.21010/Ajidv17i2S.1

**Published:** 2023-08-01

**Authors:** KONE Djakaridja, KONE Famoussa, YAPO Martine Tatiana, KADIANE-OUSSOU Juliette, KONE Salifou, KARIDIOULA Jean-Marie, KOUAME Gille Renaud, ACHO Jean Kevin, KRA Ouffoué, OUATTARA Bourheima

**Affiliations:** 1 *Department of Infectious and Tropical Diseases, University Teaching Hospital of Bouake, 01 BP 1174 Bouake 01; 2 Department of Internal Medicine, University Teaching Hospital of Bouake, 01 BP 1174 Bouake 01

**Keywords:** Aetiologies, infection, diabetes, complication, Bouake

## Abstract

**Background:**

Diabetic infections are frequent and the etiologies are multiple. The present study aims to identify the etiologies of the infections of the diabetic patient hospitalized in the University Teaching Hospital of Bouake in Côte d’Ivoire.

**Materials and Methods:**

This was a retrospective cross-sectional study conducted in the Internal Medicine Department from January 2019 to December 2020. The study population consisted of hospitalized and infected diabetic patients. Of this study population we included in the study 136 patients. Data analysis was done with Epi Info 7.2.3.1 software.

**Results:**

The prevalence of infection in hospitalized diabetics was 75.1%. The mean age of the patients was 52 ± 13.4 years. The sex ratio was 0.7. Diabetes was incidentally discovered in 50% and type 2 diabetes (88.2%) predominated. The reasons for hospitalization were dominated by ketoacidosis (58.1%), glycemic imbalance (19.1%) and hyperosmolar hyperglycemia syndrome (10.3%). Fever was present in 41.2% of cases. The infectious foci were urinary tract infections (29.4%), pneumopathies (28.7%), malaria (21.3%), skin infections (13.2%) and the undetermined focus (7.3%). The infectious focus was unique in 90.4%. The germs identified were plasmodium (21.3%), Escherichia coli (8.8%), staphylococcus (8.3%), yeasts (8.3%) and Enterobacter (6.7%). Beta-lactams (75.6%) were the most prescribed anti-infective treatment. Mortality was 14.7% related to type 1 diabetes (p=0.001), duration of diabetes greater than 5 years (p=0.005), hospitalization latency greater than 7 days (p=0.001), mucocutaneous focus (p=0.005) and Undetermined foci (p=0.001).

**Conclusion:**

Diabetic infections are frequent and the etiologies are varied. They must be systematically sought in hospitalized diabetics.

## Introduction

Diabetes mellitus is a major public health problem worldwide due to its high morbidity and mortality (International Diabetes Federation, 2012). It is a chronic metabolic disease due to disorders of glucose regulation. According to the WHO, 380 million diabetics are expected in the world by 2025, with an increase of 98.1% in developing countries (Franc, *et al* 2013). Its evolution is insidious and the discovery is often late at the stage of complications. These complications are metabolic, degenerative or infectious. Complications make treatment difficult, long and expensive (Adoueni *et al*, 2012). Infections are frequent and serious in unbalanced diabetic patients and lead to glycemic imbalance in balanced diabetic patients (Julka, 2013; Egede *et al.*, 2018; Yao *et al*, 2020). The diabetic subject is 4.4 times more exposed to the risk of infection than the non-diabetic subject with higher mortality (Burekovic *et al*, 2014). This vulnerability to infection is caused by the influence of hyperglycemia on neutrophil function, increased bacterial adhesion, decreased cytokine secretion and bacterial quiescence (Daoud *et al.*, 2009; Nikitha *et al.*, 2022). These spare no organs and frequently affect the lungs, urogenital tract, skin and soft tissues. In Côte d’Ivoire most studies on diabetes have been carried out in the city of Abidjan (Adoueni *et al.*, 2012; Kouakou *et al.*, 2016; Yao *et al*, 2020). Very few of these studies relate to the search for the infectious etiologies of diabetes in other cities of the country, hence the interest of this work which aims to identify the etiologies of infections in diabetic patients hospitalized in the University Teaching Hospital of Bouake.

### Patients and Methods

This was a retrospective cross-sectional study with descriptive and analytical purposes conducted over a period of two years; from January 1, 2019 to December 31, 2020. It took place in the Internal Medicine Department of the University Teaching Hospital (UTH) of Bouake. The UTH of Bouake is a tertiary level hospital establishment located in the center of Côte d’Ivoire. This hospital is made up of reference services receiving patients from the city of Bouake and surrounding towns in the Centre, North and West of the country. The Internal Medicine Department specializes in the management of metabolic, endocrine, tumoral, infectious and other rare diseases. It is the reference service in the care of diabetic patients in Bouake and surrounding towns. The study population consisted of adult diabetic patients hospitalized in the Internal Medicine Department in whom an infection was diagnosed. The diagnosis of the infection was retained on the basis of the presence of clinical signs (fever, signs related to a specific organ) associated or not with paraclinical signs (hyperleukocytosis, elevation of CRP, isolation of a germ, imaging signs) and favorable evolution under anti-infective treatment. All diabetic patients in whom an infection was diagnosed during the study period and who had usable medical file for the study parameters were included. A total of 136 patient records were retained. This study was carried out after obtaining authorization from the Medical and Scientific Department and the Head of the Internal Medicine Department of the Bouake University Hospital (UH). Data were collected from medical records, using a pre-established survey form, including the variables of the study: epidemiological data ( age, sex, profession, socio-economic level ), clinical data (antecedents and comorbidities, reason for hospitalization, temperature, source of infection), biological data (hemogram, CRP, thick gout, blood sugar, glycated hemoglobin, etc.), therapeutic data (anti-infectives, antidiabetics, analgesics ) and evolutionary data (duration of hospitalization, outcome of patients, cause of death). Data analysis was performed using Epi Info software version 7.2.3.1. Continuous variables were expressed as mean with standard deviation as well as extreme values and qualitative variables were expressed as proportions. Statistical comparisons were based on the chi-square test and Fischer’s test with the significance level p ≤ 0.05.

## Results

### Epidemiological aspects

Out of a total of 2457 patients hospitalized in the Internal Medicine Department during the study period, 181 were diabetic, giving a prevalence of diabetes of 7.4%. Among the 181 diabetic patients, an infection was diagnosed in 136 patients (75.1%). The mean age of the patients was 52 ± 13.4 years [18 and 85], the infection was predominant in patients aged 50 and over (62.4%). The female sex (58.9%) was predominant. The patients were uneducated in 39% of the cases, 35.3% had a craft activity, 14.7% were civil servants and 50% were unemployed.

### Clinical aspects

Diabetes was discovered during hospitalization in half of the cases (50%). Type 2 diabetes (88.2%) was predominant. Patients previously known to be diabetic were on oral (58.8%), injectable (36.8%) or mixed (4.4%) antidiabetic treatment. Patients on antidiabetic treatment were non-compliant with antidiabetic treatment in 82.4% of cases. The mean duration of their diabetes was 5.1 ± 16.5 years [1 and 15 years]. The pathological antecedents were dominated by arterial hypertension (22.8%), physical inactivity (18.4%), osteoarthritis (10.3%), HIV (3.7%) and dyslipidemia (3.7%). Hospitalization latency was less than 7 days in 86 patients (63.2%), between [7 and 21 days] in 20 patients (14.7%) and greater than 21 days in 30 patients (22.1%). The main reasons for hospitalization were ketoacidosis (58.1%), glycemic imbalance (19.1%) and hyperosmolar hyperglycemia syndrome (10.3%). The clinical symptoms were varied, dominated by fever (41.2%) as well as neurological (29.4%), digestive (25%), pleuropulmonary (24.3%), urogenital (24.3%) and mucocutaneous (11%).

### Biological aspects

Hyperglycemia was found in 98.5% of cases with an average blood sugar level of 24.97 mmol/l [8.33 and 51 mmol/l]. Glycated hemoglobin (HbA1) was greater than 7 in 96.4% of cases with an average glycated hemoglobin of 11.6 [5.5 and 16.8]. Hyperleukocytosis was found in 44.1% of patients, C-reactive protein was high in 92.3% of cases with an average of 81 mg/l [5.5 and 384]. At the microbiological level, the thick drop coupled to the blood smear was positive in 29 patients (21.3%), the parasite found was *plasmodium falciparum* in 100% of cases. The cytobacteriological and mycological examination of the urine and the blood cultures carried out made it possible to identify *Escherichia coli* (8.8%), staphylococcus (8.3%), yeasts (8.3%) and Enterobacter sp (6.7%). The etiologies found are listed in Table I.

**Table 1:** distribution of patients according to etiology

Variable	Proportion	Percentage
**Number of hearths**		
1	123	90.4
2	12	8.8
3	01	0.8

**Infectious foci**		
Urogenital	40	29.4
Pneumonia	39	28.7
Malaria	28	20.6
mucocutaneous	18	13.2
Undetermined foci	10	7.3
Digestive	4	2.9
Meningitis	2	1.5
Cellulite	2	1.5
Toxoplasmosis	1	0.7
Gonarthritis	1	0.7

### Therapeutic aspects

Antibiotics were the most prescribed anti-infective treatment with, in order of frequency, beta-lactams (75.6%), fluoroquinolones (29.4%) and aminoglycosides (23.5%) followed by antimalarials (21 .3%).

### Evolutionary aspects

Mortality was 14.7% and the causes of death were dominated by septic shock (65%) and hypoglycemia (20%). In univariate analysis, the factors associated with death were type 1 diabetes (p=0.001), hospitalization latency greater than 7 days (p=0.001), duration of diabetes greater than 5 years (p=0.005), mucocutaneous infections (p=0.005) and Undetermined foci (p=0.001). (Table 2).

**Table 2:** Factors associated with the death of hospitalized patients.

Sociodemographic parameters	Death	Healing	P value
**Age (years)**			
Age < 50	12	85	
Age ≥ 50	8	31	**0.89**

**Sex**			
Male	6	51	
Feminine	14	65	**0.85**

**Clinical parameters**			

**type of diabetes**			
Type 1	2	11	
Type 2	18	105	**0.001**

**Duration of diabetes (years)**			
Inaugural	9	59	**0.6**
≥ 5	7	13	**0.005**
< 5	4	44	**0.19**
**Hospitalization latency (days)**			
< 7	13	73	**0.85**
[7-21]	3	17	**0.001**
>21	4	26	**0.001**

**Type of infection**			
Malaria	3	25	**0.71**
Pneumonia	6	33	**0.88**
mucocutaneous	6	12	**0.005**
Urogenital	7	33	**0.3**
Undetermined foci	1	9	**0.001**
Meningitis	1	1	**0.17**

**Number of infectious foci**			
1	19	104	**0.11**
Several	1	12	

## Discussion

This study allowed the identification of aetiologies of infections in diabetic subjects hospitalized in the internal medicine department of the UTH of Bouake. The limitations of this study are related to its retrospective nature, but the data collected was usable and allowed us to produce relevant results.

In this study, prevalence of infection in hospitalized diabetic patients was 75.1%. This high frequency of infection of the diabetic subject would be the consequence of a reduction in the response of T lymphocytes, an alteration in the function of polymorphonuclear neutrophils and disorders of humoral immunity (Julka, *et al*, 2013; Burekovic *et al*, 2014). Therefore, diabetes mellitus increases susceptibility to infections. The frequency of infection in diabetics generally remains high in hospitalization, so infection is one of the main causes of glycemic imbalance (Gupta, *et al.*, 2007; Burekovic, *et al.*, 2014). The mean age of the patients was 52 ± 13.4 years. The average age of onset of diabetes is 55 years, several authors have reported an interval of discovery of diabetes between 40 and 65 years (Sayadi *et al.*, 2015; Kouakou *et al.*, 2016; Yao *et al.*, 2020). This average age reflects the predominance of type 2 diabetes, which occurs in subjects over 40 years old (Mokhtar *et al.*, 2009). In our study, the majority of patients were over 60 years old. The elderly diabetic subject is more susceptible to infections due to the aging of the immune defense system, malnutrition, prostatic hypertrophy constituting an obstacle on the urinary tract in men (Gninkoun *et al.*, 2019).

The female sex was predominant (58.7%) with a sex ratio of 0.7. This female predominance has been reported by some authors (Daoud *et al.*, 2009; Yao *et al.*, 2017), on the other hand other authors have reported a male predominance (Ouedraogo *et al.*, 2000; Kouakou *et al.*, 2016). This gender discrepancy would be due to the methodology deployed in the study and depends on the socio-demographic characteristics of the population studied. In this study, the predominance of female hospitalized diabetics would be due to the high cost of diabetes care for a predominantly poor female population, unemployed and without income. This situation favors the delay of consultation and would be at the origin of the occurrence of several complications, in particular infectious, which require hospitalization. Type 2 diabetes (88.2%) was the majority, as reported by several authors in developing and Western countries (Adoueni *et al.*, 2012; International Diabetes Federation *et al.*, 2012; Franc*, et al.*, 2013). The high prevalence of type 2 diabetes is due to the modification of dietary habits and the increase in cardiovascular risk factors in the population (Mokhtar *et al.*, 2009). The infection revealed diabetes in half of the patients in the study, this is due to the ignorance of the population and the lack of education of the populations in the face of early detection of diabetes.

In Côte d’Ivoire as in all developing countries, there is no policy of early detection and follow-up of diabetic patients, diabetes is most often revealed during a complication (Adoueni *et al.*, 2012; Kouakou *et al.*, 2016). The general signs and the signs related to the affected organs guided the prescription of the biological and radiological assessment in order to establish the diagnosis. The rate of completion of the etiological work-up was low due to the high cost of the analysis which were difficult to carry out by patients who were mostly poor and without health insurance (Adoueni *et al.*, 2012).

The germs identified were *plasmodium falciparum* (21.3%), *Escherichia coli* (8.1%), staphylococcus (5.1%), enterobacter (3%) and yeasts (2.9%). The infectious foci found were varied, dominated by urogenital infections, pneumopathies, malaria and sepsis. The infectious sites remain the same with a distribution variously reported in the literature. Like the reports of Burekovic (2014) and Sayadi (2015), we have a predominance of urogenital and pulmonary infections. *Plasmodium falciparum, Escherichia coli*, staphylococcus, yeasts and enterobacter sp were the most common germs in diabetic patients, this corresponds to the ecology of germs usually encountered in our regions (Gninkoun *et al.*, 2019; Yao *et al.*, 2020). The high mortality in this study would reflect the poverty and ignorance of the populations. Indeed, the treatment of diabetes is totally the responsibility of the diabetic in our developing countries, they most often resort to self-medication or traditional therapy which is less expensive than hospital treatment. All these promote delays in consultation and non-compliance with antidiabetic treatment. Diabetics are most often seen at an advanced stage of their disease or the prognosis is poor (Adoueni *et al.*, 2012).

## Conclusions

This study was carried out to enrich the information concerning the etiologies of infections in diabetic subjects at the Bouake University Hospital. Analysis of the results shows that the prevalence of infections remains high in diabetics in the Internal Medicine Department of the Bouake University Hospital. Infections occur in type 2 diabetic subjects, with an average age of 52 years; and in females living in unfavorable socioeconomic conditions. The clinical symptomatology is dominated by fever and the cardinal signs of diabetes. Infections were dominated by urinary tract infections, pneumopathies, malaria, mucocutaneous infections and sepsis. The germs found were *plasmodium falciparum, Escherichia coli*, staphylococcus, Enterobacter and yeasts. Mortality was high in connection with type 1 diabetes, duration of diabetes greater than 5 years, latency of hospitalization greater than 7 days, mucocutaneous focus and sepsis.

### Conflicts of interest

The authors declare that there is no conflict of interest associated with this study.

**List of Abbreviations:**
E. Coli: Escherichia Coli
CRP: C-reactive protein
IDF: International Diabetes Federation
OAD: oral antidiabetic
Staph Aureus: *Staphylococcus Aureus*
T lymphocytes: Thymus lymphocytes
UHC: University Hospital Center
UTH: University Teaching Hospital
WHO: World Health Organization.
